# Building Research Competence Across a Nursing Program: A Descriptive Documentary Study

**DOI:** 10.3390/nursrep16050168

**Published:** 2026-05-15

**Authors:** Lucília Nunes, Andreia Ferreri Cerqueira, Ana Poeira

**Affiliations:** 1 Escola Superior de Saúde, Instituto Politécnico de Setúbal, 2910-761 Setubal, Portugal; andreia.cerqueira@ess.ips.pt (A.F.C.); ana.poeira@ess.ips.pt (A.P.); 2 Comprehensive Health Research Centre [CHRC]—Polo NMS, 1169-056 Lisboa, Portugal

**Keywords:** undergraduate students, nursing education, evidence-based practice, active learning, learning outcome

## Abstract

The organized integration of research competencies into nursing curricula is still a global challenge and is key for preparing professionals to respond to complex clinical contexts, technological advancements, and contemporary societal demands. At the School of Health of the Polytechnic Institute of Setúbal, a longitudinal research axis was implemented across the four years of the undergraduate nursing program, involving epistemological foundations, the research process, evidence-based practice, and applied practice. **Objective:** The objective of this study was to describe the design and implementation of the longitudinal axis of research, analyzing institutional indicators of academic success and the progressive development of students’ scientific competencies. **Methods:** A descriptive documentary study based on institutional data analysis (the number of enrolled students, pass rates, and mean grades in the four research-related curricular units) was conducted, complemented by a review of pedagogical materials produced (two published course booklets: “Research I—From the origin to the dissemination of knowledge” and “Research II—(De)Constructing the Research Process: A Critical and Practical Analysis”) and evidence of scientific dissemination (conference presentations and published articles). **Results:** A continuous progression in academic performance was observed across the research curricular units, accompanied by increased complexity of student work and enhanced scientific literacy. The sequential structure proved essential: the articulation of epistemology, methodology, critical appraisal, and scientific production demonstrated strong coherence and pedagogical efficiency. **Conclusions:** The longitudinal research axis constitutes a curricular innovation that strengthens essential scientific competencies in undergraduate nursing education. Longitudinal models that reflect both conceptual and practical progression can significantly contribute to the development of nurses who are critical thinkers, reflective practitioners, and capable of integrating evidence into clinical decision-making.

## 1. Introduction

The increasing complexity of healthcare systems, rapid technological development, and the growing emphasis on quality and safety in care delivery have reinforced the central role of research competence in nursing practice. Contemporary nurses are expected not only to provide clinically sound care but also to critically appraise scientific evidence, integrate research findings into decision-making, and contribute to the continuous improvement of healthcare services. Evidence suggests that competencies such as evidence-based practice, communication, and clinical reasoning are interrelated and should be developed in an integrated manner during nursing education [1]. In addition, structured educational strategies are essential to support the development of evidence-based practice competencies among nursing students across both academic and clinical settings [2]. Consequently, the development of research competence has become a core objective of undergraduate nursing education worldwide.

Despite this recognition, the systematic integration of research education into nursing curricula, especially through student-centered methods, remains a global challenge due to factors such as limited time, task ambiguity, unequal workload, and insufficient guidance [3]. Research-related content is often concentrated in isolated curricular units, introduced late in the program, or taught primarily as a set of technical skills disconnected from clinical reasoning and professional practice [4,5,6]. Such fragmented approaches may limit students’ ability to develop scientific literacy over time and to view research as an integral component of nursing practice rather than an abstract academic requirement [7,8].

The educational literature increasingly supports longitudinal and scaffolded curricular models as efficient strategies for promoting complex competencies, including critical thinking, evidence-based practice, and professional autonomy [9]. In nursing education, longitudinal research pathways allow students to gradually engage with epistemological foundations, methodological reasoning, critical appraisal of evidence, and scientific dissemination, promoting deeper learning and sustained competence development [7,8,10,11]. However, empirical descriptions of such integrated curricular models, particularly at the undergraduate level, remain limited.

At the School of Health of the Polytechnic Institute of Setúbal (Portugal), a longitudinal research axis was intentionally designed and implemented across the four years of the undergraduate nursing program. This axis articulates epistemology, the research process, evidence-based nursing, and applied research, thereby safeguarding continuity and increasing cognitive and methodological complexity. Rather than treating research as a peripheral or isolated domain, the program positions scientific research as a foundational and transversal element of nursing education.

The longitudinal research axis is operationalized through four mandatory curricular units (Research I to Research IV), each with clearly defined learning objectives, pedagogical strategies, and assessment methods that build explicitly on prior learning. The axis is further supported by the production of pedagogical materials and by learning activities that emphasize critical reflection, ethical awareness, and the translation of evidence into practice.

The present study aims to describe the design and implementation of this longitudinal research axis and to analyze its educational impact using institutional academic indicators and pedagogical outputs. By presenting this curricular model, the study seeks to support the ongoing discussion on innovative approaches to research education in undergraduate nursing programs and to offer a transferable framework for institutions aiming to strengthen scientific competence and evidence-based practice among future nurses.

## 2. Context and Design of the Longitudinal Research Axis

The undergraduate Nursing Program at the School of Health of the Polytechnic Institute of Setúbal (Portugal) has implemented a longitudinal research axis designed to progressively develop students’ scientific literacy and research competence throughout the four years of the program. This curricular axis addresses well-documented challenges in nursing education, including fragmented approaches to research and teaching, delayed exposure to evidence-based practice, and difficulties translating scientific knowledge into clinical decision-making.

Rather than concentrating research education in a single curricular unit, the program adopts a structured, sequential model in which research-related competencies are introduced, consolidated, and applied across four mandatory curricular units (Research I–IV). This longitudinal design ensures epistemological coherence, methodological continuity, and increasing cognitive and practical complexity, supporting students’ progressive engagement in scientific research.

### 2.1. Pedagogical Rationale and Curricular Coherence

The design of the research axis is grounded in the assumption that research competence in nursing requires more than technical methodological training [12]. It involves understanding the nature of scientific knowledge, critical reasoning, ethical awareness, and the ability to apply evidence in real clinical contexts. Consequently, the axis was conceived as a scaffolded pedagogical pathway in which each curricular unit explicitly builds on the knowledge and skills developed in the previous one.

This progression aligns with contemporary perspectives on competency-based education and evidence-based nursing, emphasizing the integration of epistemological foundations, methodological rigor, critical appraisal, and scientific dissemination as core professional competencies [11,12,13,14].

### 2.2. Structure of the Longitudinal Research Axis

The longitudinal research axis is composed of four sequential curricular units distributed across the undergraduate program (Table 1):

Research I—Nursing Epistemology (1st year)

This unit introduces students to the epistemological foundations of science and nursing knowledge. It addresses nature, sources, limits, and validity of scientific knowledge, exploring key epistemological theories and their relevance to nursing as a scientific discipline. Emphasis is placed on scientific literacy, knowledge paradigms, and the relationship between science, research, and professional practice. Pedagogical strategies include conceptual analysis, critical reflection, and engagement with foundational texts, fostering early familiarity with scientific thinking.

Research II—Research Process (2nd year)

Building on the epistemological foundations established in Research I, this unit focuses on the systematic understanding of the research process. Students explore the conceptual, methodological, empirical, and dissemination phases of research, with particular attention to critical analysis of scientific articles. Ethical principles are addressed transversally. The unit adopts a strongly applied approach, combining theoretical explanation with practical exercises and critical appraisal of published articles, enabling students to understand how methodological choices influence the quality of evidence.

Research III—Evidence-Based Nursing (3rd year)

In the third year, the focus shifts from understanding research to actively using research to inform practice. This unit focuses on evidence-based nursing and the development of competencies in searching, selecting, appraising, and synthesizing scientific evidence. Students are introduced to systematic reviews of the literature using the Joanna Briggs Institute methodology and progress through the data extraction phase. The unit emphasizes clinical relevance, methodological rigor, and structured synthesis of evidence, bridging research and professional decision-making.

Research IV—Research Project (4th year)

The final unit consolidates the research pathway by guiding students to complete a research project grounded in evidence synthesis. Building on the work initiated in Research III, students advance through the remaining stages of the review process, with a strong focus on analysis, synthesis, and discussion of findings, as well as increasing responsibility for scientific dissemination. Students’ progress from data extraction to qualitative and quantitative synthesis, ethical analysis of secondary studies, and scientific writing. Emphasis is placed on dissemination strategies, including the preparation of research reports and scientific articles. Alumni participation and seminar-based activities further reinforce professional identity as evidence-informed practitioners.

**Table 1:** Longitudinal research axis.

Characteristic	Research I	Research II	Research III	Research IV
Curricular Year	1st year	2nd year	3rd year	4th year
Focus	Nursing epistemology and scientific thinking	Research design and critical appraisal	Evidence-based nursing	Evidence synthesis and scientific dissemination
Key Activities	Conceptual analysis and epistemological reflection	Critical appraisal of the scientific literature	Methodology of systematic reviews	Analysis, synthesis, discussion, and scientific dissemination
Emphasis	Scientific literacy and epistemological foundations	Ethical principles and methodological reasoning	Clinical relevance and methodological rigor	Dissemination strategies and professional identity

This pedagogical progression also aligns with the competency profile expected of general care nurses, particularly in the domains of professional development, quality improvement, and evidence-based decision-making. These competencies are framed within broader professional standards that emphasize the integration of scientific knowledge into clinical practice and the continuous improvement of care.

In the first year (Research I), students develop foundational competencies in scientific thinking, epistemological awareness, and an understanding of the nature and limits of knowledge. These competencies support the development of professional responsibility and critical reflection, which are essential for ethical and informed decision-making in nursing practice.

In the second year (Research II), students acquire competencies in understanding the research process, including research design, methodological reasoning, and the critical appraisal of scientific studies. These skills contribute to the development of analytical thinking and the ability to assess the quality and applicability of evidence, supporting safe and informed care delivery.

In the third year (Research III), the focus shifts toward applying evidence-based practice competencies. Students learn to formulate clinical questions, conduct systematic searches, critically appraise evidence, and interpret findings in light of clinical relevance. These competencies are directly aligned with the nurse’s role in promoting quality of care, improving outcomes, and supporting evidence-informed decision-making.

In the fourth year (Research IV), students consolidate their competencies through evidence synthesis, scientific writing, and dissemination of research findings. At this stage, students demonstrate greater autonomy, contributing to knowledge production and reflecting competencies in professional development, lifelong learning, and improving healthcare practices.

By explicitly linking curricular activities to professional competencies, the longitudinal research axis ensures that research education is not perceived as an isolated academic requirement but as a core dimension of nursing practice, reinforcing the integration between scientific knowledge and clinical decision-making.

These competencies are developed through a combination of theoretical and practical learning activities. Each curricular unit includes structured contact hours combining lectures, guided practice, and student-led activities, ensuring consistent exposure to research-related content throughout the semester.

Active learning strategies are systematically employed, including critical appraisal exercises, structured group work, case-based discussions, guided analysis of scientific articles, and seminar-based presentations. In the third and fourth years, research activities are developed in small groups, allowing distribution of workload and fostering collaborative learning. The systematic review process is initiated in Research III and further developed in Research IV, ensuring a progressive and manageable workload across both years. These activities are embedded within the core curricular units and aligned with assessment methods, rather than being offered as additional or extracurricular components.

The intensity and complexity of these activities increase progressively over the four years, with earlier stages focusing on guided learning and later stages promoting greater autonomy, collaboration, and scientific production.

### 2.3. Pedagogical Materials as Evidence of Implementation

The implementation of the longitudinal research axis is supported by the development of dedicated pedagogical materials produced by the teaching team (Table 2). Two course booklets were published as open-access educational resources: “Research I—From the Origin to the Dissemination of Knowledge” [15] and “Research II—(De)Constructing the Research Process: A Critical and Practical Analysis” [16]. These materials reflect the axis’s curricular philosophy, combining theoretical frameworks, practical examples, guided exercises, and critical reflection.

These booklets cover the full scope of the curricular content for the respective units and are designed to support the research pathway along the longitudinal axis. They are not intended as comprehensive longitudinal records of all student activities or as a full “journey portfolio” across the program. Instead, they function as structured pedagogical resources that ensure alignment between learning objectives, teaching strategies, and assessment methods across the research-related curricular units. Beyond their instructional function, these booklets serve as tangible scholarly outputs that document the pedagogical design and contribute to the model’s sustainability and transferability.

As an extension of the work developed in Research III and Research IV, multiple knowledge dissemination outputs have emerged, including presentations at scientific events and peer-reviewed publications (Table 2). These outputs reflect students’ progressive engagement with evidence synthesis, critical appraisal, and scientific writing, illustrating the translation of curricular learning into scholarly production. Importantly, several of these publications are later integrated into the curriculum as pedagogical resources, supporting peer learning and enabling subsequent cohorts to engage with authentic examples of student-led scientific work. Refs. [17,18,19] illustrate some of the scientific dissemination outcomes resulting from this research pathway.

These dissemination outputs represent selected examples of student work and are not expected from all students.

### 2.4. Summary of the Longitudinal Design

Overall, the longitudinal research axis represents a curricular innovation that integrates epistemology, methodology, evidence appraisal, and scientific communication into a coherent educational pathway. By progressively increasing complexity and autonomy, the axis aims to prepare undergraduate nursing students not only to understand research, but to critically engage with evidence and apply it meaningfully in clinical practice.

## 3. Materials and Methods

A descriptive documentary study was conducted to examine the design, implementation, and educational impact of a longitudinal research axis integrated into an undergraduate nursing program. This methodological approach was considered appropriate given the study’s focus on curricular structure, pedagogical materials, and aggregated institutional academic indicators, rather than on individual-level outcomes or experimental comparisons.

### 3.1. Context of the Study

The study was conducted at the School of Health of the Polytechnic Institute of Setúbal (Portugal) within the undergraduate Nursing Program. The program includes a longitudinal research axis distributed across four mandatory curricular units (Research I, Research II, Research III, and Research IV), implemented sequentially over the four years of the degree.

The research axis aims to promote the progressive development of research competence, scientific literacy, and evidence-based practice through structured pedagogical progression from epistemological foundations to applied research and scientific dissemination.

### 3.2. Data Sources

Institutional academic indicators, obtained from internal records, include aggregated data on the number of enrolled students, pass rates, and mean final grades for the research-related curricular units.

### 3.3. Data Analysis

Institutional academic indicators were analyzed using descriptive statistics, which allowed the identification of trends in academic performance across curricular units and academic years. No inferential statistical analyses were conducted, as the study did not aim to establish causal relationships. To minimize potential sources of bias, the study used aggregated institutional data covering all students enrolled in the research-related curricular units across three academic years, avoiding sampling bias. Data were obtained from official institutional records to ensure consistency and accuracy of measurement.

### 3.4. Ethical Considerations

The study was based exclusively on aggregated institutional data and publicly available pedagogical materials. No individual student records were accessed, and no personal or identifiable data were used. According to institutional and national guidelines, ethical committee approval was not required for this type of documentary and educational analysis.

## 4. Results

To analyze student performance and progression in research competencies, aggregated academic indicators from the research-related curricular units (Research I, Research II, Research III, and Research IV) were examined across three academic years. The indicators analyzed included the number of enrolled students, assessed students, and students who passed each course unit, as well as mean grade, standard deviation, and minimum–maximum grades. Assessment data refer to continuous assessment results. The number of assessed students includes all students with a final grade, including those who failed, whereas mean, standard deviation, and minimum–maximum grades were calculated based on students who passed the course unit. These indicators were used to explore trends in academic performance and student success across the different stages of the research training pathway. In Portugal, higher education grading is based on a 0–20 scale, with 9.5 as the minimum passing grade. For reporting purposes, grades ≥ 10 were considered approved.

The results show high success rates across all research-related curricular units and academic years. A progressive increase in mean grades is observed from Research I and II to Research III and IV, suggesting a positive trend in academic performance across the program. These findings may be consistent with the progressive development of research-related competencies; however, these indicators should be interpreted as indirect measures (Table 3). Lower mean grades and higher standard deviations observed in the earlier curricular units may reflect students’ initial exposure to research methodology and scientific thinking, whereas higher, more homogeneous grades in Research IV may indicate consolidation of research competencies and greater student autonomy in conducting research-related tasks (Table 3).

A decrease in the number of enrolled students, namely in Research III, in the academic year of 2024–2025 is explained by institutional mobility and double-degree pathways. A group of 10 students enrolled in an international double-degree nursing program completed equivalent research training modules, thereby granting equivalence to the Research III curricular unit. Therefore, the reduction in the number of students enrolled in this curricular unit does not reflect dropout or academic failure, but rather participation in an alternative curricular pathway within the program’s internationalization strategy.

The analysis of the academic performance indicators for the longitudinal research axis (Research I–IV) between 2022 and 2025 shows four major trends:

(1) Upward performance trajectory, because there is a consistent increase in mean grades as students progress from Research I and II to Research III and IV; this suggests that the curriculum is consistent with research competencies over time, leading to higher achievement in the more advanced stages of the longitudinal axis.
(2) High success, because we can see the conversion from “Assessed” to “Passed” is remarkably high, often reaching 100% in the final units (Research III and IV); while there is a natural “drop-off” in enrollment (enrolled vs. assessed) in the initial units, those who reach the final stages of the research track show near-perfect pass rates.
(3) Increased group homogeneity, because the standard deviation (SD) tends to decrease significantly by the time students reach Research IV; this indicates that the student cohort becomes more uniform in their excellence. The “gap” between the highest and lowest performers closes as they master research methodologies.
(4) Continuous improvement in foundational units, meaning that there is a positive trend specifically in Research I; looking to the mean grade in those three years (14.3, 15.67, and 16.9), this suggests institutional improvements in how research is introduced, or a cohort of students entering the program with stronger baseline skills each year.

## 5. Discussion

This study aimed to describe the design and implementation of a longitudinal research axis integrated into an undergraduate nursing program. Longitudinal approaches in educational research provide a valuable framework for understanding how learning processes may evolve over time, particularly when curricular structures are intentionally designed to support progressive development. Within this perspective, the findings of the present study contribute to the understanding of how a structured and coherent curricular model may be associated with positive academic trends across different stages of training.

The findings of this study suggest that a longitudinal, structured research curriculum may support the progressive development of research competence, scientific literacy, and evidence-based practice skills among undergraduate nursing students, who must demonstrate these skills and should be highlighted in a longitudinal program to highlight the outcomes [7,11,20,21]. Understanding learning trajectories allows teachers (and researchers) to move beyond measuring simple pre-test/post-test gains and instead model continuous growth trajectories, identifying whether student progress is linear, non-linear, accelerating, or decelerating.

The progressive increase in mean grades across the research-related curricular units appears to reflect not only academic progression but also increasing cognitive complexity, methodological autonomy, and engagement with scientific work.

These results can be interpreted in light of scaffolded learning theory, which suggests that complex competencies are more effectively developed when learning is structured progressively, with increasing levels of difficulty and autonomy [9,22,23]. Rather than concentrating on research education in a single curricular moment, the longitudinal structure allowed students to revisit research concepts over time, integrating epistemological reflection, methodological reasoning, critical appraisal, and scientific dissemination. This progressive exposure may facilitate deeper learning and the development of higher-order cognitive skills, such as critical thinking and scientific reasoning [24,25,26]. By following participants over time, teachers (and researchers) can better determine whether specific teaching strategies or interventions lead to improved learning outcomes, or whether improvements in engagement, such as through longitudinal, spiraled curriculum design, result in higher achievement.

The integration of research education across the curriculum also supports the development of evidence-based practice competencies. Previous studies have shown that students often struggle to connect research methodology with clinical decision-making when research is taught as an isolated subject [1,23,25,27]. By integrating epistemology, research methods, and evidence-based practice into a coherent pathway, the longitudinal model may help students view research as a practical and professional tool rather than an abstract academic requirement.

Also, repeated data collection can create longitudinal records that reveal, for instance, that a student who disengages after a turbulent start is at high risk of low achievement, allowing for timely, targeted interventions. This is especially evident in the transition between the third and fourth years (Research III and IV).

An important aspect of this model is its contribution to the development of professional identity as evidence-informed practitioners. Professional identity formation in nursing is a developmental process that occurs throughout education and is influenced by learning experiences that integrate knowledge, practice, and reflection [28]. As students progress from understanding the nature of knowledge to producing and disseminating research, they begin to perceive themselves not only as consumers of knowledge but also as potential contributors to scientific development in nursing, which is consistent with research-based education approaches that position students as active participants in knowledge production. This shift may be particularly important for strengthening the scientific identity of the nursing profession and reinforcing nurses’ role as evidence-informed practitioners [29,30].

The existence of international mobility and double-degree pathways that require equivalence with research-related curricular units also suggests that the longitudinal research axis is sufficiently structured and coherent to enable curricular integration across institutions, reinforcing its transferability and curricular robustness.

It is important to emphasize that academic performance indicators, such as grades and pass rates, do not directly measure research competence. Rather, they reflect student performance within specific assessment contexts and may be influenced by multiple factors, including assessment design, teaching strategies, and student characteristics. Therefore, the findings should be interpreted cautiously, as indicators of trends consistent with competence development rather than as definitive evidence of such development.

This study has some limitations. The study is based on aggregated institutional data and does not include individual-level measures of research competence, critical thinking, or evidence-based practice skills. In addition, academic grades may reflect multiple factors, including assessment methods and student characteristics, and should therefore be interpreted as indirect indicators of competence development. Future studies could include qualitative data, student perceptions, or competency assessment tools to better understand the impact of longitudinal research education.

### Implications for Nursing Education

The results of this study have several implications for undergraduate nursing education. First, they underscore the importance of introducing research education early in the curriculum, grounded in epistemological reflection and scientific reasoning, rather than delaying research-related learning until later stages of training. Early exposure may foster students’ understanding of research as a foundational element of professional nursing practice.

Second, the longitudinal and scaffolded structure of the research axis demonstrates how progressive curricular design can support the development of complex competencies, such as critical appraisal of evidence and application of research findings to clinical decision-making. Nursing programs seeking to strengthen evidence-based practice may benefit from adopting integrated models that ensure continuity and increasing levels of autonomy across curricular units.

Third, developing pedagogical materials aligned with curricular objectives is a key strategy for sustaining educational innovation. Such resources can enhance consistency across teaching teams, support student learning, and serve as documented evidence of pedagogical intent and curricular alignment.

Finally, this study highlights the value of institutional reflection on curricular design as an educational scholarship. Systematic documentation and analysis of curricular innovations can help disseminate effective educational practices and inform future developments in nursing education. Related to our study are several key reasons for the reinforcement of research in nursing education, such as enabling students to integrate the best current evidence with clinical expertise and patients’ values, reduce errors, and improve quality care. It is necessary to enhance professionals’ ability to analyze complex situations and find solutions to clinical questions and situations in other contexts because students should also develop analytical skills necessary for leadership, policy development, and adapting to new technologies like AI in healthcare.

## 6. Conclusions

This study presents the design and implementation of a longitudinal research axis integrated across the four years of an undergraduate nursing program. By articulating epistemological foundations, the research process, evidence-based practice, and scientific dissemination, the axis provides a coherent and progressive framework for developing research competence in nursing education. The analysis of institutional academic indicators and pedagogical outputs suggests that a structured and longitudinal curricular approach may support the progressive development of scientific literacy, critical thinking, and research-related competencies among undergraduate nursing students. The findings also highlight the importance of curricular continuity and constructive alignment between learning objectives, teaching strategies, and assessment methods. Research competence in nursing is not developed in a single course, but through a longitudinal educational process that integrates epistemology, methodology, evidence appraisal, and scientific dissemination over time. This type of curricular model may be transferable to other nursing programs and health-related disciplines seeking to strengthen research competence and evidence-based practice in higher education.

## Figures and Tables

**Table 2:** Pedagogical materials and scholarly outputs within the longitudinal research axis.

Pedagogical Dimension	Course Booklets	Knowledge Dissemination Outputs
Purpose	Instructional and scholarly educational resources	Scholarly and pedagogical dissemination of student-produced knowledge
Content	Theoretical frameworks, practical examples, guided exercises, and critical reflection	Evidence synthesis, critical appraisal, scientific writing, and research dissemination
Impact	Document pedagogical design and support curricular sustainability and transferability	Support peer learning and provide authentic scholarly exemplars for subsequent cohorts
Examples	Open-access course booklets (Research I; Research II)	Conference presentations and peer-reviewed publications

**Table 3:** Academic performance indicators across the longitudinal research curricular units (Research I–IV), 2022–2025.

Academic Year	Curricular Unit	Enrolled (n)	Assessed (n)	Passed (n)	Mean Grade	SD	Min–Max
2022–2023	Research I	61	55	51	14.3	1.69	11–18
	Research II	52	50	48	14.8	1.71	11–18
	Research III	46	45	45	17.7	1.10	14–19
	Research IV	44	44	44	18.4	0.61	17–19
2023–2024	Research I	51	44	44	15.67	1.02	14–18
	Research II	50	48	44	14.2	1.11	12–16
	Research III	46	46	45	16.8	1.69	10–18
	Research IV	44	44	44	18.1	1.01	16–19
2024–2025	Research I	46	46	46	16.9	0.75	15–19
	Research II	45	38	38	14.7	1.79	12–18
	Research III	36	34	34	16.7	2.52	11–19
	Research IV	41	41	41	17.4	1.04	16–19

Data refer to continuous assessment results. Overall success rates for the curricular units may be higher, as some students complete the course during the final examination period.

## Data Availability

No new data were created or analyzed in this study. Data sharing is not applicable to this article.

## Public Involvement Statement

There is no public involvement in any aspect of this research.

## Guidelines and Standards Statement

This study was reported in accordance with the STROBE guidelines.

## Abbreviations

The following abbreviations are used in this manuscript:

CHRC	Comprehensive Health Research Centre
SD	Standard Deviation
Min	Minimum Grade
Max	Maximum Grade
AI	Artificial Intelligence
